# In silico profiling of cell growth and succinate production in *Escherichia coli* NZN111

**DOI:** 10.1186/s40643-016-0125-5

**Published:** 2016-11-15

**Authors:** Xingxing Jian, Ningchuan Li, Cheng Zhang, Qiang Hua

**Affiliations:** 1 State Key Laboratory of Bioreactor Engineering, East China University of Science and Technology, Shanghai, China; 2 Science for Life Laboratory, KTH-Royal Institute of Technology, Stockholm, Sweden; 3 Shanghai Collaborative Innovation Center for Biomanufacturing Technology (SCICBT), Shanghai, China

**Keywords:** NZN111, Succinate, NADH/NAD^+^, Artificial centering hit-and-run (ACHR), Sample solution space, Metabolite flux-sum analysis

## Abstract

**Background:**

Succinic acid is a valuable product due to its wide-ranging utilities. To improve succinate production and reduce by-products formation, *Escherichia coli* NZN111 was constructed by insertional inactivation of lactate dehydrogenase (LDH) and pyruvate formate lyase (PFL) encoded by the genes *ldhA* and *pflB*, respectively. However, this double-deletion mutant is incapable of anaerobically growing on glucose in rich or minimal medium even with acetate supplementation. A widespread hold view is that the inactivation of NADH-dependent LDH limits the regeneration of NAD^+^ and consequently disables proper growth under anaerobic conditions.

**Results:**

In this study, genome-scale metabolic core model of *E. coli* was reconstructed and employed to perform all simulations in silico according to the reconstruction of engineered strain *E. coli* NZN111. Non-optimized artificial centering hit-and-run (ACHR) method and metabolite flux-sum analysis were utilized to evaluate metabolic characteristics of strains. Thus, metabolic characteristics of the strains wild-type *E. coli*, *ldhA* mutant, *pflB* mutant, and NZN111 under anaerobic conditions were successfully unraveled.

**Conclusions:**

We found a viewpoint contrary to the widespread realization that an NADH/NAD^+^ in NZN111 mainly resulted from the inactivation of PFL rather than the inactivation of LDH. In addition, the two alternative anaerobic fermentation pathways, lactate and ethanol production pathways, were blocked owing to the disruption of *ldhA* and *pflB*, resulting in insufficient NAD^+^ regeneration to oxidize or metabolize glucose for cell growth. Furthermore, we speculated reaction NADH16, the conversion of ubiquinone-8 (q8) to ubiquinol-8 (q8h2), as a potential amplification target for anaerobically improving cell growth and succinate production in NZN111.

**Electronic supplementary material:**

The online version of this article (doi:10.1186/s40643-016-0125-5) contains supplementary material, which is available to authorized users.

## Background

Succinic acid is a commodity of immense commercial value and has wide-ranging utilities in chemical, food, and pharmaceutical industries (Ahn et al. 2016). In addition, the U.S. Department of Energy (DOE) has listed succinic acid among the top 12 bio-based building block chemicals that can be produced by microbial fermentation (Werpy et al. 2004). At present, several metabolically engineered microorganisms, including *Saccharomyces cerevisiae*, *Escherichia coli*, and *Mannheimia succiniciproducens,* are employed to produce succinate. Under anaerobic conditions, wild-type *E. coli* brings about mixed-acid fermentation but produces only minor quantities of succinate. Therefore, to overcome this limitation, *E. coli* NZN111 was constructed by insertional disruption of the *ldhA* and *pflB* genes, which encode the lactate dehydrogenase (LDH) and pyruvate formate lyase (PFL) enzymes, respectively, to improve succinate production and reduce by-product formation (Matjan et al. 1989; Bunch et al. 1997). However, this double-deletion mutant is unable to anaerobically metabolize glucose in rich or minimal medium, even after supplementing with acetate (Lucy Stols MID 1997; Bunch et al. 1997). A widely hold view is that the inactivation of the NADH-dependent LDH limits the regeneration of NAD^+^ and consequently hinders growth under anaerobic conditions (Ahn et al. 2016). Reports suggest that decreasing the intracellular redox ratio (NADH/NAD^+^) in NZN111 improved growth and succinate production (Singh et al. 2009). Overexpression of malate dehydrogenase (MDH), encoded by the gene *mdh*, in NZN111 restored its ability to anaerobically metabolize glucose due to favorable regeneration of NAD^+^ from NADH and redox balance (Wang et al. 2009). A spontaneous mutant AFP111 (NZN111 *ptsG* ^−^) has also been shown to exhibit cell growth on glucose and improved anaerobic succinate production with suppressed normal glucose repression (Chatterjee et al. 2001). In addition, an increase of succinate yield and a decrease of pyruvate accumulation in *E. coli* NZN111 (Wu et al. 2007) and its derivative AFP111 (Jiang et al. 2010) were observed using a dual-phase fermentation mode, where a high cell density was achieved in the first aerobic growth phase, and subsequently succinate was produced in the second anaerobic production phase.

Therefore, we can observe that the imbalance of the redox ratio (insufficient regeneration of NAD^+^ from NADH) in NZN111 resulted in poor growth under anaerobic conditions. However, it is not clear whether the insufficient regeneration of NAD^+^ from NADH is a result of LDH or PFL inactivation in NZN111. It is well recognized that *E. coli* possesses a robust regulatory catabolic system, which allows it to cope with a wide range of environmental or genetic perturbations (Ishii et al. 2007). For continued cell growth, it is crucial that NADH be oxidized to NAD^+^ and a redox balance be achieved (Berrios-Rivera et al. 2002b). *E. coli* can grow either in the presence of an electron acceptor (such as oxygen, nitrate, fumarate) or fully fermentatively. However, under anaerobic conditions and in the absence of an alternate oxidizing agent, the regeneration of NAD^+^ is achieved by using NADH to reduce metabolic intermediates. For example, pyruvate can be reduced to lactate or ethanol along with the regeneration of NAD^+^ from NADH (Zhu and Shimizu 2004).

In recent decades, genome-scale metabolic models (GEMs) have been utilized to better understand the genotype–phenotype relationship in microbial metabolism (Famili et al. 2003; Lewis et al. 2012; Matsuoka and Shimizu 2015) and suggest manipulation strategies for strain design (Burgard et al. 2003; Gu et al. 2016; Jian et al. 2016). Meanwhile, a steady-state flux space is defined, which contains all possible functional states due to the constraint-based reconstructed network (Orth et al. 2010; Schellenberger et al. 2011). The inherent uncertainty in constraint-based models is well suited to study by employing Monte Carlo sampling (Schellenberger and Palsson 2009). Uniform random sampling of the steady-state flux space allows for an unbiased and detailed assessment of the impact of the applied physicochemical constraints on a reconstructed network (Papin et al. 2004; Price et al. 2004). For example, the probability distributions of the values of individual metabolic flux showed a wide variety of shapes, and the pairwise correlation coefficients were calculated between all fluxes, determining the level of independence between any two fluxes and identifying the highly correlated reaction sets. Although global network properties are of interest, sampling has been successfully applied to study clinical issues (Jamshidi and Palsson 2006; Occhipinti et al. 2007; Thiele et al. 2005).

In the present study, an unbiased artificial centering hit-and-run (ACHR) method (Kaufman and Smith 1998), one improvement to the Monte Carlo sampling, was utilized to sample the solution space and illustrate the global network properties of the constraint-based models. In addition, metabolite flux-sum analysis (Chung and Lee 2009) was utilized to calculate global turnovers of several cofactors, which aided in understanding the physiological metabolism of microorganisms (Lakshmanan et al. 2015a, b). Therein, genome-scale metabolic core models of *E. coli* were employed to perform all simulations in silico. We sampled the solution space of the wild-type *E. coli*, *ldhA* mutant, *pflB* mutant, and NZN111 using ACHR method to characterize their respective network properties. Subsequently, metabolic characteristics of these strains with respect to cell growth and succinate production under anaerobic conditions were successfully unraveled. On the basis of metabolite flux-sum analysis, we also found a viewpoint opposed to the widespread realization that in NZN111, NADH/NAD^+^ imbalance mainly resulted from the inactivation of PFL rather than the inactivation of LDH, which was consistent with the experimental results and gene expression profile of the wild-type *E. coli* and NZN111. In addition, the two alternative anaerobic fermentation pathways (Berrios-Rivera et al. 2002b; Yang et al. 1999a; Yun et al. 2005), including lactate and ethanol production pathways, were blocked owing to the disruption of *ldhA* and *pflB*, resulting in insufficient NAD^+^ regeneration to oxidize or metabolize glucose for cell growth. Furthermore, the analysis of metabolic characteristics and correlations suggested that strengthening reaction NADH16 could contribute towards the improvement of cell growth and succinate production in NZN111 under anaerobic conditions. Overall, this work provides us a new in silico insight into the physiological metabolism of microorganisms.

## Methods

### The constraint-based models

Genome-scale metabolic core models of *E. coli* downloaded from BiGG database were employed to perform all simulations in silico (Schellenberger et al. 2010). Here, the relation of reactant and product in some reactions was exchanged to simulate positive flux values without affecting the correlation between reactions. The output was termed **Model** for the wild-type *E. coli* in this study (Additional file 1). *E. coli* NZN111 was created by insertional inactivation of LDH and PFL encoded by *ldhA* and *pflB*, respectively, to improve succinate production and reduce by-product formation. Strain NZN111 produced very low levels of lactate and formate in LB medium (Lucy Stols MID 1997) or M9 medium (Wang et al. 2009) under anaerobic conditions. Additionally, in LB medium *ldhA* mutant produced little lactate, while *pflB* mutant also produced little formate (Lucy Stols MID 1997; Zhu and Shimizu 2004). Accordingly, we obtained the modified model for *E. coli* NZN111 by directly deleting reactions LDH-D and PFL regulated by *ldhA* and *pflB*, respectively, named **Model_nzn**. Similarly, deleting reactions LDH-D or PFL from **Model** were constructed for *ldhA* mutant or *pflB* mutant, respectively, called **Model_del_ldh** and **Model_del_pfl**.

### Artificial centering hit-and-run algorithm

Artificial centering hit-and-run (Kaufman and Smith 1998), an unbiased random walk algorithm, was employed to randomly sample the steady-state flux space and shape the solution space, which illustrated the global network properties of constraint-based models or reflected the physiological metabolism of microorganisms under specific conditions (Kaufman and Smith 1998; Occhipinti et al. 2007; Schellenberger and Palsson 2009; Thiele et al. 2005). This algorithm involves three steps: The first step requires the identification of an initial point that lies within the solution space but not at the extremity. The second step of ACHR calculates “warm-up” points from the initial point using several iterations of a basic hit-and-run algorithm. The warm-up points are stored as columns of a matrix ***W***, and an approximate center, ***s***, is calculated. In the final step, the sample points are calculated. The direction for the next iteration from a sample point ***x***_***m***_ is chosen by randomly taking one point ***y*** out of the matrix ***W*** and applying the direction vector of ***y*** and ***s*** (${\vec{\text{y}}}$−${\vec{\text{s}}}$ ) to ***x***_***m***_. At each iteration, the newly calculated point, ***x***_***m*+*1***_, was substituted randomly into ***W*** in the place of a previously calculated point. The approximate center was also recalculated following iteration. This last step is repeated until a desired number of sample points are reached.

In the present study, we sampled the solution space of the constraint-based models using default settings by loading 2000 points per reaction, i.e. 2000 flux values per reaction were provided for subsequent analysis. Therefore, an unbiased average flux value could be obtained by calculating the average value and standard deviation of each reaction (Almaas et al. 2004), and a histogram for each reaction could be plotted to illustrate the probability distributions of flux values. Moreover, Pearson’s correlations between reactions can be calculated as well.

### Metabolite flux-sum analysis

A descriptive variable of flux-sum was defined to represent the turnover rate of a metabolite by summing up all the incoming or outgoing fluxes around the metabolite at quasi-steady state (Chung and Lee 2009). This definition clearly indicates that the unit of flux-sum is equivalent to that of the reaction flux (i.e., mmol/g DW/h). Here, the concept of flux-sum was applied to the average flux distributions that resulted from the samplings of quasi-steady-state flux space using unbiased ACHR algorithm. Still, we let *Φ*_*i*_ denote the flux-sum of metabolite *i*, and its mathematical form is given by *Φ*_*i*_ = 0.5 $\mathop \sum \nolimits_{j} \left| {S_{ij} v_{j} } \right|$. Metabolite flux-sum analysis elucidates the roles of metabolites in the network. In addition, a change of overall flux-sum of metabolites reflects an alteration of the metabolic network with perturbation. Analysis of the overall turnovers of cofactors (e.g. NAD(P)H) can aid in understanding the roles of the identified genes (or enzymes) (Lakshmanan et al. 2015a, b).

### Reaction expression profile

Gene expression data of wild-type *E. coli* BW25113 and NZN111 strains were downloaded from the GEO database (http://ftp.ncbi.nlm.nih.gov/pub/geo/DATA/supplementary/samples/GSM546nnn/GSM546949/GSM546949.CEL.gz). Cells of the wild-type *E. coli* BW25113 and NZN111 strains growing microaerobically in M9 medium containing 10 g/L glucose were harvested in the mid-exponential phase. Total RNA was isolated using Qiagen’s RNeasy kit following the manufacturer’s protocol. Expression data of 136 genes existed in **Model** except for gene *s0001* were selected for preprocessing using log2 (Additional file 2). Subsequently, a two-sample Kolmogorov–Smirnov test was performed to compare the two groups of values, viz. the gene expression data for wild-type *E. coli* and NZN111 strains. Results of the two-sample test showed statistically significant differences (p value = 1.6348 × 10^−13^; at 5% significance level) between the two *E. coli* strains.

Furthermore, reaction expression profile can be mathematically characterized by gene expression data based on logical relations of gene reaction described in the model as follows: 

Thus, the ratios of logarithmic reaction expression data for strain NZN111 to that of wild-type *E. coli* were calculated to better indicate the differential expression reactions.

## Results and discussion

### *In silico* simulation and analysis of the effects of *ldhA* and *pflB* knockout

**Fig. 1:**
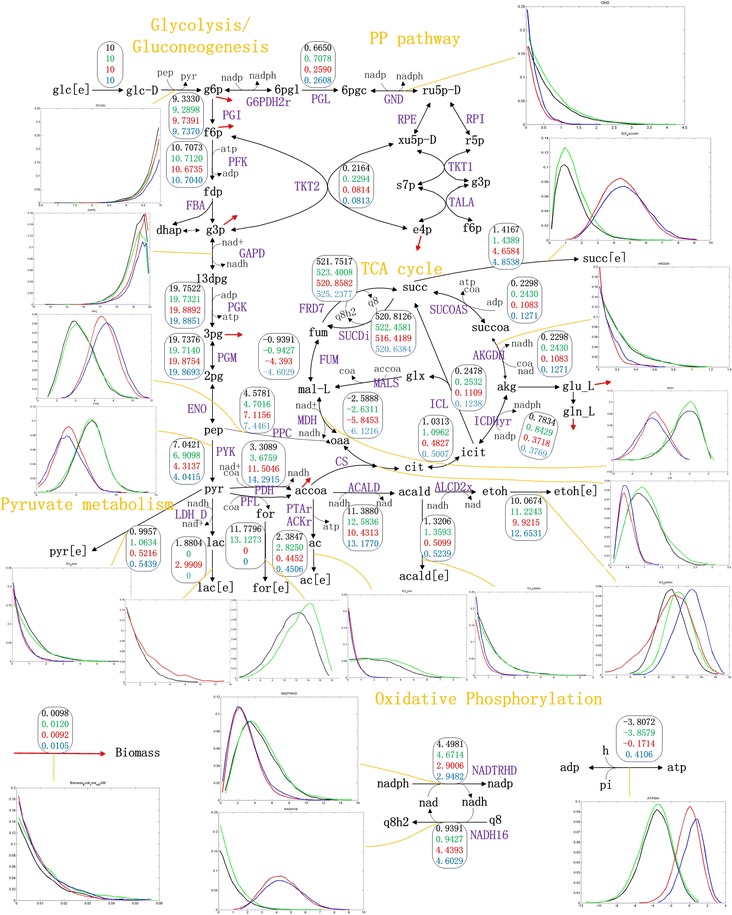
Metabolic flux distributions simulated by non-optimized ACHR method under anaerobic conditions. The abbreviations and *arrows in black color* represent metabolites and reactions, respectively, whereas the* red arrows* refer to the biomass formation reaction. The *rectangles* and *histograms* denote the average flux values of reactions and the probability distributions for the values of reactions, respectively. Therein, the colors of both the *numbers in the rectangles* and the *lines in the histograms* indicate different strains as follows: *black*, *green*, *red*, and *blue* indicate wild-type *E. coli*, *ldhA* mutant, *pflB* mutant, and NZN111, respectively

*Escherichia coli* NZN111 was constructed to anaerobically produce succinate by the disruption of *ldhA* and *pflB* genes. Accordingly, based on the metabolic core model of *E. coli*, we constructed four constraint-based models: **Model**, **Model_del_ldh**, **Model_del_pfl**, and **Model_nzn** for wild-type *E. coli*, *ldhA* mutant, *pflB* mutant, and NZN111, respectively (see “Methods” section). These models were employed to randomly sample the solution space using the ACHR method. The sampling errors encountered during the procedure were minimal (<1 × 10^−6^), indicating the correctness of these sampling results. On the basis of these sampling results, we calculated the average flux and standard deviation of each reaction and plotted a histogram for each reaction (Additional files 3, 4). Therefore, the average flux distributions consisted of average fluxes for wild-type *E. coli*, *ldhA* mutant, *pflB* mutant, and NZN111 were achieved to shape their global metabolisms. And each histogram illustrated the probability distribution of the values of each reaction. Furthermore, for the purpose of convenient comparison, the four average flux distributions were normalized to the specific glucose uptake rate of 10 mmol/g DW/h (Additional file 3) (Fig. 1). Therefore, the metabolite flux-sum values of cofactors on the basis of the four normalized flux distributions can be evaluated as shown in Table 1.

**Table 1:** Metabolic characteristics of several models, including fluxes of several exchange reactions and flux-sum values of several key cofactors. (unit: mmol/g DW/h)

	Model	Model_del_ldh	Model_del_pfl	Model_nzn
Biomass	0.0098	0.0120	0.0092	0.0105
EX_succ (e)	1.4167	1.4389	4.6584	4.8538
EX_lac_D (e)	1.8804	0	2.9909	0
EX_for (e)	11.7796	13.1273	0	0
EX_pyr (e)	0.9957	1.0634	0.5216	0.5439
EX_acald (e)	1.3206	1.3593	0.5099	0.5239
EX_ac (e)	2.3847	2.8250	0.4452	0.4506
EX_etoh (e)	10.0674	11.2243	9.9215	12.6531
Atp flux-sum	29.4088	29.7098	24.7564	24.9150
Ctp flux-sum	0	0	0	0
Fadh2 flux-sum	0	0	0	0
Nadh flux-sum	28.7541	29.3178	35.1824	38.1361
Nadph flux-sum	5.5522	5.7709	3.8235	3.9129
Q8h2 flux-sum	521.7517	523.4008	520.8582	525.2377
Accoa flux-sum	15.0885	16.8031	11.5046	14.2915

**Fig. 2:**
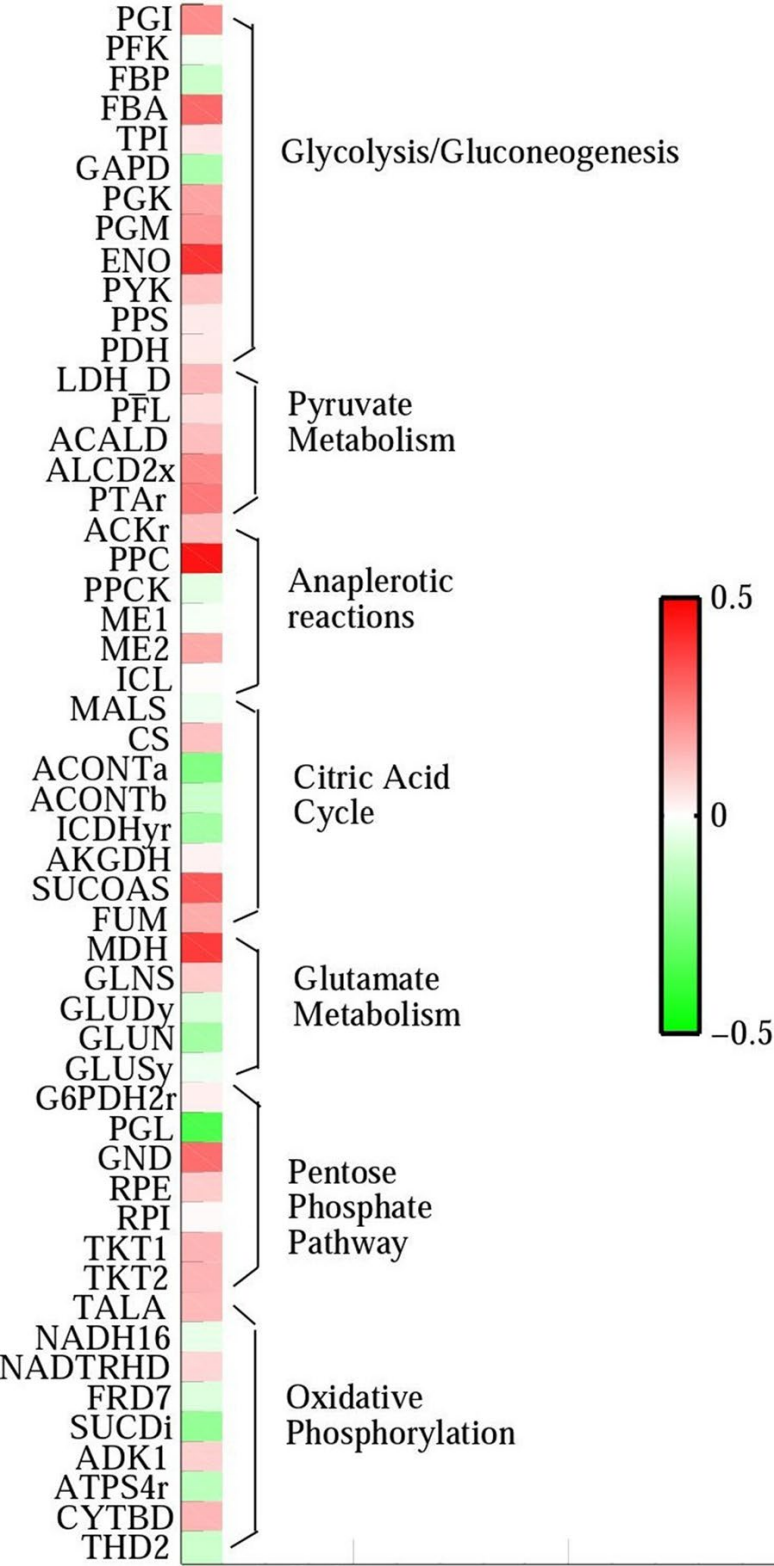
Heat map plotted to illustrate the ratios of logarithmic reaction expression data for strain NZN111 to those for wild-type *E. coli* minus one, which are represented as a gradient of colors from *green* (−0.5) to *red*  (+0.5). The reactions are ordered by metabolic pathways

Figure 1 shows that the carbon source for producing succinate under anaerobic conditions is mostly derived from phosphoenolpyruvate (pep) pool through the reductive TCA pathway in all four models. Respective deletion of reaction LDH-D or PFL all increased the specific production rate of succinate and reduced the biosynthesis of by-products (Table 1). In strain NZN111, simultaneous deletion of reactions LDH-D and PFL enforced carbon flow towards the reversed TCA cycle via reaction PPC catalyzed by phosphoenolpyruvate carboxylase encoded by the gene *ppc* and reduced biosynthesis of by-products such as lactate, acetaldehyde, acetate, and ethanol. Similarly, researchers have validated that strengthening the reductive TCA pathway by overexpressing the *ppc* gene enabled succinate as a major product in *E. coli* under anaerobic conditions (Millard et al. 1996). In addition, the results from our simulations were also consistent with the gene expression data of wild-type *E. coli* BW25113 and NZN111. Compared to the wild-type strain, expression data of reactions PPC, MDH, and FUM in strain NZN111 indicate significant up-regulation (Fig. 2; Additional file 2).

Furthermore, in strain NZN111, a high ratio of NADH/NAD^+^ (imbalanced redox state) resulted in growth defects on glucose under anaerobic conditions. (Singh et al. 2009). In addition, a high flux-sum value of NADH was found in **Model_nzn** (Table 1), which we considered to be mainly a result of knockout of reaction PFL. The reason for this assumption was that the knockout of reaction PFL caused a much higher flux-sum value of NADH than that caused by knockout of reaction LDH-D, although knockout of reaction LDH-D resulted in a slightly enhanced flux-sum value of NADH as well. The quantities of increased NADH turnover resulting from the inactivation of PFL were about 11-fold higher than that of inactivation of NADH-dependent LDH. That is to say that in strain NZN111 a high ratio of NADH/NAD^+^ may mainly result from the inactivation of PFL rather than the inactivation of LDH, which can probably be attributed to the two NAD^+^ formed from ethanol production versus one NAD^+^ from lactate formation for each molecule of pyruvate (Yang et al. 1999b).

The ethanol and lactate production pathways are two of the most robust anaerobic fermentative pathways in *E. coli* for the regeneration of NAD^+^ from NADH for cell growth. (Zhu and Shimizu 2004). Experimental results have shown that the anaerobic cell growth is not affected by deficiency of the *ldh* gene, while the *pfl* mutant could only grow in glucose medium supplemented with acetate (Graef et al. 1999; Lucy Stols MID 1997). Numerous studies investigated the two fermentative pathways that are utilized to regenerate NAD^+^ from NADH in *E. coli*. For example, overexpression of the NAD^+^-dependent formate dehydrogenase in *E. coli* resulted in a significant redistribution of metabolic fluxes, as evidenced by a dramatic increase in the ethanol-to-acetate ratio and a decrease in the flux to lactate (Berrios-Rivera et al. 2002a). In addition, the experimental results have also shown that *ackA*-*pta* mutant has a reduced acetate level and a much higher lactate formation rate with simultaneously lower formate and ethanol synthesis rates. The observed diversion of flux to lactate is mainly due to the necessity of NAD^+^ regeneration (Yang et al. 1999a). Moreover, a mutant with severely impaired ethanol production under anaerobic conditions was found to have generally improved the production of lactate and succinate (Yun et al. 2005). Thus, we concluded that lactate and ethanol production pathways can be an alternate way to regenerate NAD^+^ from NADH in order to maintain a proper redox balance of cofactor NADH/NAD^+^. However, in strain NZN111, the inactivation of LDH and PFL due to the disruption of genes *ldhA* and *pflB*, respectively, blocked the two alternate pathways and failed to regenerate sufficient NAD^+^ to oxidize glucose under anaerobic conditions.

### *In silico* simulation and analysis of succinate accumulation in *E. coli* NZN111

**Fig. 3:**
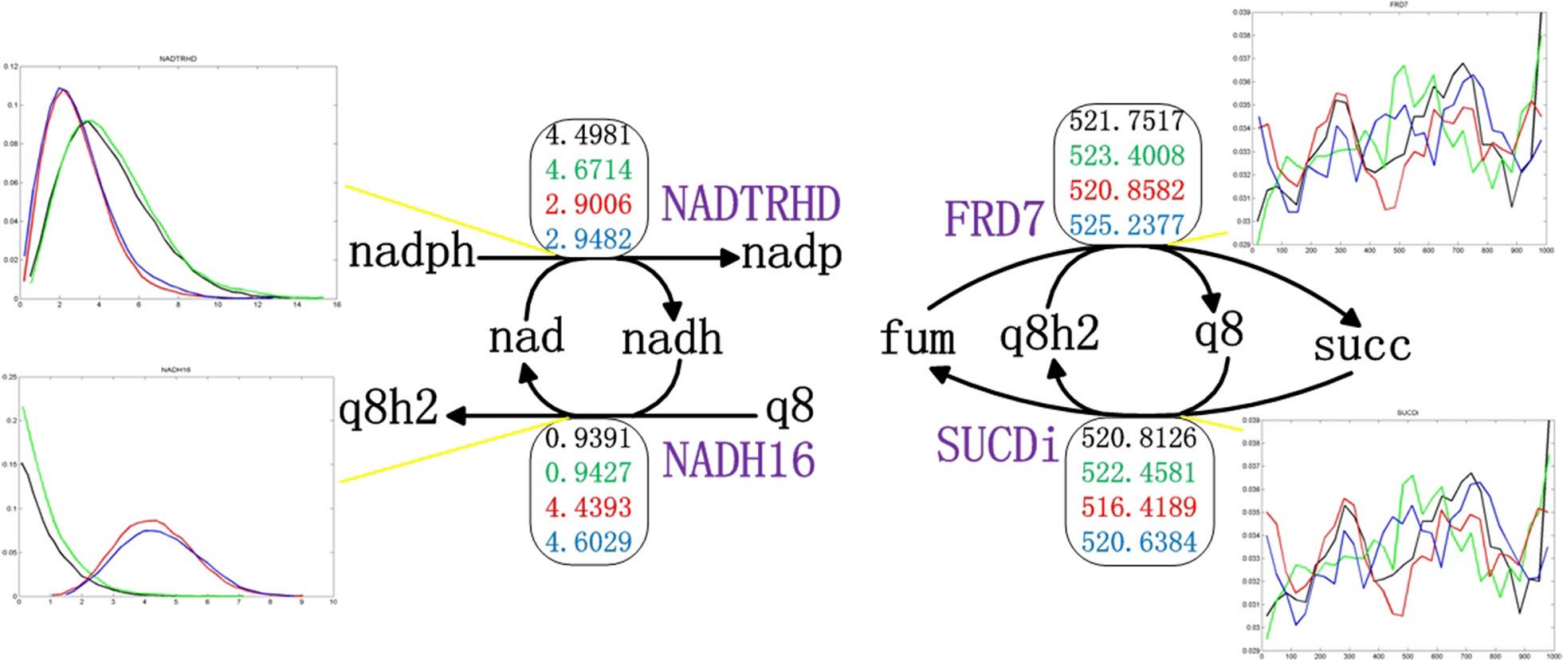
Two loops to couple NADH/NAD^+^ and succinate production under anaerobic conditions. The *abbreviations* and *arrows in black color* represent metabolites and reactions, respectively. The *abbreviations in purple* refer to the reaction names. The *rectangles* and *histograms* denote the average flux values of reactions and the probability of reactions’ fluxes, respectively. Therein, the colors of both the numbers in the *rectangles* and the *lines in the histograms* stand for different strains: *black*, *green*, *red*, and *blue* indicate the wild-type *E. coli*, *ldhA* mutant, *pflB* mutant, and NZN111, respectively

Additionally, we found that the accumulated NADH enforced the conversion of ubiquinone-8 (q8) to ubiquinol-8 (q8h2) by strengthening reaction NADH16, while q8h2 facilitated the conversion of fumarate to succinate (Fig. 3). This observation was consistent with the gene expression data of the wild-type *E. coli* BW25113 and NZN111. Compared to the wild-type strain, gene expression of reaction NADH16 was significantly up-regulated and that of reaction NADTRHD was significantly down-regulated (Fig. 2). Thus, we speculated that strengthening reaction NADH16 by overexpressing the corresponding genes may be an effective way not only for decreasing the intracellular ratio of NADH/NAD^+^ but also for enhancing succinate production. Reaction NADH16 is catalyzed by enzyme complexes encoded by the *nuoA*-*N* gene cluster (14 subunits), which encode a transmembrane NADH:ubiquinone oxidoreductase (NDH-I) responsible for coupling redox chemistry to proton-motive force generation (Yang et al. 1999b). Singh et al. (2009) reported that overexpressing *nuoC* coding for the connecting fragment of the NDH-I (1 subunit of NDH-I) effectively decreased NADH/NAD^+^ ratio in the cell and improved cell growth and succinate production under anaerobic conditions.

### *In silico* simulating correlation of succinate production in *E. coli* NZN111

**Fig. 4:**
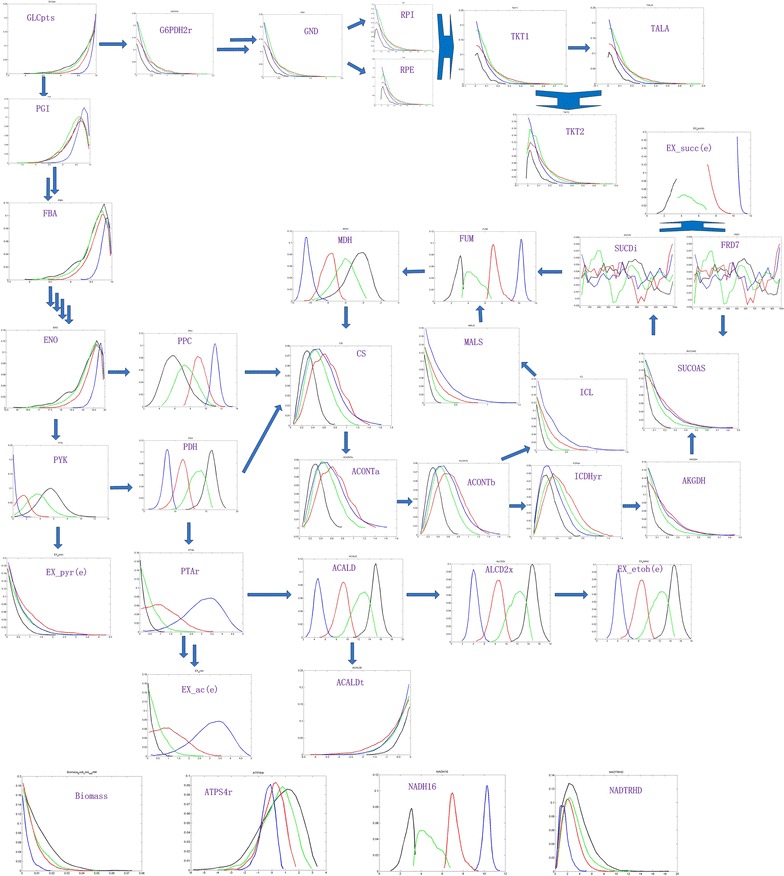
Metabolic flux distributions simulated by non-optimized ACHR method under anaerobic conditions. The *arrow* refers to the flow direction, while the *purple abbreviations* refer to the reaction names. Each *histogram* denotes a reaction, which represents the probability distributions of the values of reaction. Therein, the *colors* of the *lines* in each *histogram* indicate different strains: *black*, *green*, *red*, and *blue* indicate the wild-type *E. coli*, *ldhA* mutant, *pflB* mutant, and NZN111, respectively

To simulate what correlates to cell growth and succinate production in *E. coli* NZN111 under anaerobic conditions, **Model_nzn** stepwise setting distinct upper and lower bounds of succinate exchange reaction [i.e., reaction EX_succ(e)] were performed to sample the solution space using ACHR method. Meanwhile, histograms were plotted and the normalized average flux distributions were evaluated as described above (Additional files 5, 6). As with the elevated specific production rate of succinate [i.e., the fluxes of reaction EX_succ(e) gradually elevated], more carbon source flow towards the TCA cycle via reaction PPC and less acetyl-coA synthesis was apparent (Fig. 4; Table 2). Meanwhile, the higher the accumulation of NADH, the smaller the specific growth rate was, *i.e.,* the accumulated NADH inhibited cell growth. However, the flux-sum value of NADH gradually decreased because accumulated NADH was used to facilitate the regeneration of q8h2, which also improved succinate production. As shown above, the accumulation of NADH facilitated reaction NADH16 and restrained reaction NADTRHD, thus decreasing the turnover of NADH and NADPH gradually (Table 2).

**Table 2:** Metabolic characteristics of several models, including fluxes of several exchange reactions and flux-sum values of several key cofactors. (unit: mmol/g DW/h)

	Model_nzn_A	Model_nzn _B	Model_nzn _C	Model_nzn _D
Bounds	[0, 3.4764]	[3.4764, 6.9529]	[6.9529, 10.4293]	[10.4293, 13.9058]
Biomass	0.0119	0.0085	0.0071	0.0036
EX_succ (e)	2.9783	5.0800	7.6984	10.7240
atp flux-sum	26.4170	24.6409	22.8017	23.5086
ctp flux-sum	0	0	0	0
fadh2 flux-sum	0	0	0	0
nadh flux-sum	40.8157	37.9489	34.3208	30.4042
nadph flux-sum	4.5915	4.0034	3.1865	1.8573
q8h2 flux-sum	515.0937	514.8774	509.5948	511.4309
accoa flux-sum	16.3295	13.9790	11.2263	8.6963

**Fig. 5:**
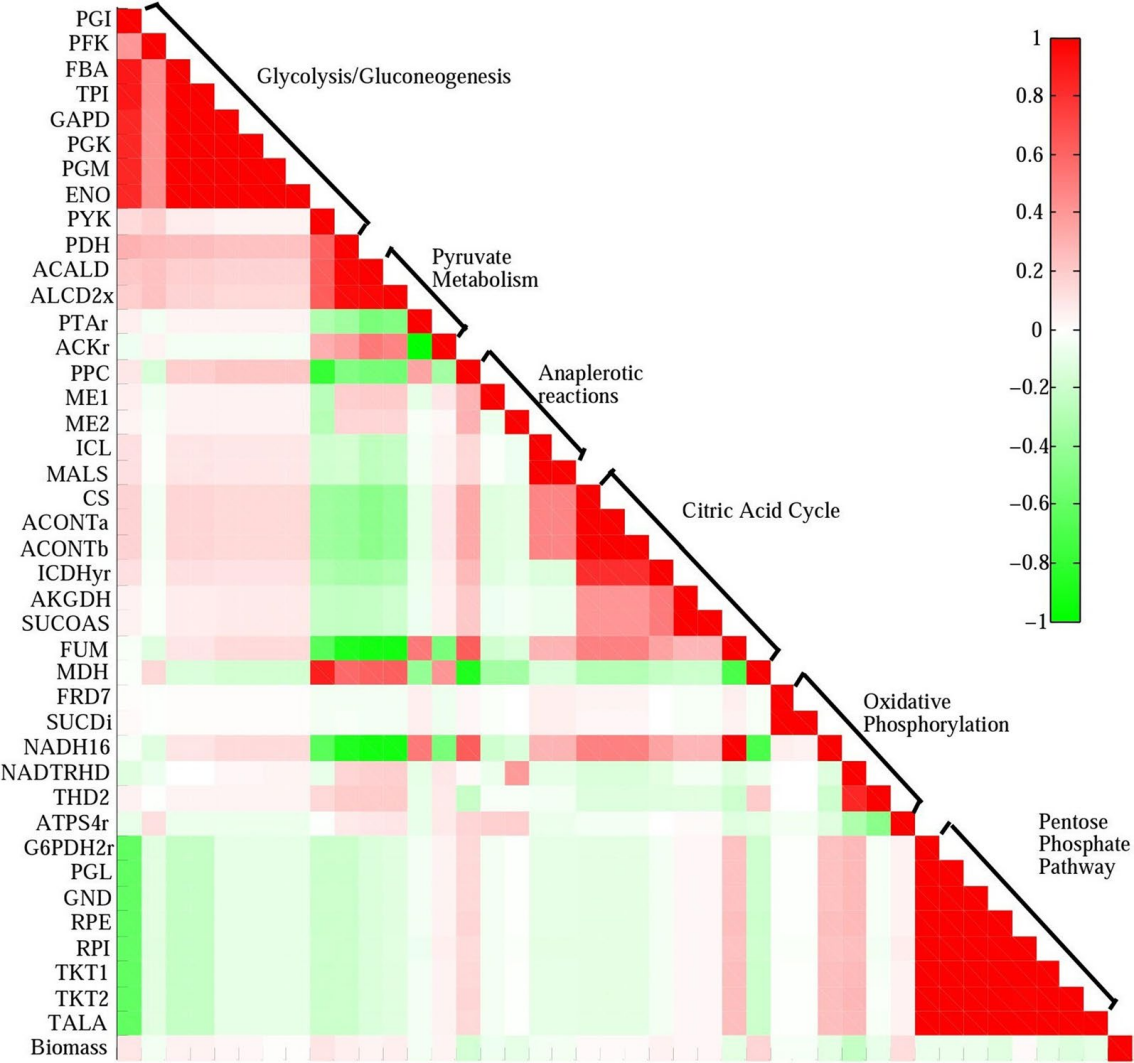
Matrix of Pearson’s correlations between reactions in **Model_nzn**. Pearson’s correlation values between each pair of fluxes are represented as a gradient of colors from *green* (−1) to *red* (+1). The fluxes are ordered by metabolic pathways

Additionally, based on the 2000 fluxes for each reaction obtained using the ACHR method in **Model_nzn** [without constraint to reaction EX_succ(e)], a matrix of Pearson’s correlations between reactions was calculated and is shown in Fig. 5. Reaction NADH16 negatively correlated with pyruvate metabolism (ACALD, ALCD2x) because of NADH consumption, *i.e.,* inactivation of pyruvate metabolism (ACALD, ALCD2x) due to the disruption of *ldhA* and *pflB* genes suppressing the biosynthesis of acetyl-coA facilitated reaction NADH16 to convert q8 to q8h2. This further confirms that strengthening reaction NADH16 may be an effective strategy for decreasing accumulation of NADH and increasing succinate production in strain NZN111.

Similarly, reaction NADH16 negatively correlated with reaction MDH. In addition, cell growth was found to be positively correlated with reaction MDH due to regeneration of NAD^+^, and with reaction ATPS4r due to the supply of ATP. Moreover, researchers reported that overexpressing malate dehydrogenase (MDH) encoded by the gene *mdh* improved cell growth and succinate production in *E. coli* NZN111 (Wang et al. 2009). Furthermore, overexpression of ATP-forming PEPCK from *Actinobacillus succinogenes* in an *ldhA* ^−^, *pflB* ^−^, *ptsG* ^−^, and *ppc* ^−^ quadruple mutant strain resulted in a 60% increase in biomass and succinate formation (Singh et al. 2011).

## Conclusions

In this study, non-optimized artificial centering hit-and-run (ACHR) algorithm was utilized to sample the solution space, and metabolite flux-sum analysis was utilized to calculate global turnovers of several cofactors. The use of such methods allowed us to successfully unravel the metabolic networks of cell growth and succinate production under anaerobic conditions. In contrast to the widespread consensus that the imbalance of NADH/NAD^+^ mainly resulted from the inactivation of PFL rather than that of LDH in strain NZN111, our studies show a different mechanism. Results from the present study demonstrated that the disruption of *pflB* gene limits the regeneration of NAD^+^ to a greater extent compared to the disruption of *ldhA* gene in strain NZN111. These results are also consistent with the experimental results and gene expression profiles of wild-type and NZN111 strains of *E. coli*. In addition, the two alternative anaerobic fermentation pathways, namely the lactate and ethanol production pathways, were also blocked owing to the disruption of the *ldhA* and *pflB* genes, resulting in insufficient NAD^+^ regeneration to oxidize or metabolize glucose for cell growth.

Additionally, on the basis of the analysis of metabolic characteristics and correlations, we speculated that strengthening reaction NADH16 may be an effective target for improving cell growth and succinate production in strain NZN111 under anaerobic conditions.

In summary, using an unbiased non-optimized ACHR method allows for the sampling of solution space of constraint-based models, which can shape the global network properties and provide insights into the physiological metabolism of microorganisms under specific conditions.

## Additional files

**Additional file 1.** Modified metabolic core model of *E. coli*.

**Additional file 2.** Gene expression data.

**Additional file 3.** The metabolic flux distributions for the wild-type *E .coli* and its mutants.

**Additional file 4.** Reactions histograms for the wild-type *E.coli*, *ldhA* mutant, *pflB* mutant and NZN111.

**Additional file 5.** The metabolic flux distributions for *E. coli* NZN111 at distinct upper and lower bounds of reaction EX_succ(e).

**Additional file 6.** Reactions histograms for NZN111 at distinct upper and lower bounds of reaction EX_succ(e).
